# Technical Feasibility of Custom-Fabricated Auxetic Foam Insoles for Plantar Pressure Redistribution: An Exploratory Pilot and Bootstrap Resampling Investigation

**DOI:** 10.3390/ma19163446

**Published:** 2026-08-14

**Authors:** LaBreesha Batey, Enrique M. Jackson, Changchun Zeng, Selvum Pillay

**Affiliations:** 1 Department of Mechanical and Materials Engineering, The University of Alabama at Birmingham, 1075 13th St S, Birmingham, AL 35294, USA; pillay@uab.edu; 2 Human Landing System Branch, George C. Marshall Space Flight Center, National Aeronautics and Space Administration, 4631 Saturn RD, Huntsville, AL 35812, USA; 3 Material Science Branch, George C. Marshall Space Flight Center, National Aeronautics and Space Administration, 4631 Saturn RD, Huntsville, AL 35812, USA; enrique.m.jackson@nasa.gov; 4 Department of Industrial and Manufacturing Engineering, FAMU-FSU College of Engineering, Florida Agricultural and Mechanical University, 2525 Pottsdamer St., Tallahassee, FL 32310, USA; zeng@eng.famu.fsu.edu

**Keywords:** auxetic materials, re-entrant foam, plantar pressure redistribution, peripheral neuropathy, biomechanical engineering, custom orthotics

## Abstract

**Highlights:**

- Custom auxetic foam insoles demonstrate exploratory propensity for plantar pressure mitigation across diverse neuropathic subsystems.
- Bootstrap analysis (*B* = 1000) indicates structural load consistency with a simulated standard deviation of 12.063 kPa.
- Etiology-specific customization reduces peak dynamic pressure by up to 62.2% in systemic polyneuropathies within this pilot cohort.
- Volumetric custom inserts can trigger a localized crowding effect that elevates pressure trends in focal mononeuropathies.
- MARVUE 2D kinematic tracking reveals qualitative trends toward stabilized gait paths and consistent roll-through cycles.

**Abstract:**

Peripheral neuropathy degrades gait mechanics, elevating peak plantar pressures (PPP) and tissue ulceration risks. This exploratory pilot study evaluates custom orthotics using thermo-mechanically synthesized re-entrant auxetic foam insoles with targeted dome-shaped inserts. Bilateral dynamic gait analysis was conducted across a heterogeneous cohort (*N* = 9) utilizing a P-Walk 600 pressure plate and a MARVUE 2D motion capture system inside standardized footwear. To address small-sample limits, a non-parametric bootstrap resampling analysis (*B* = 1000) was executed. Native auxetic foam demonstrated high internal consistency ((σ*) = 12.063 kPa, Consistency Rank = 1), showing a strong numerical propensity to restrict load distribution variability compared to factory-installed over-the-counter (OTC) memory foam controls. While initial cell-adaptation cycles were observed, custom insoles reduced PPP by up to 62.2% in systemic polyneuropathies, shifting signatures beneath the exploratory clinical safety target (<200 kPa) in 55% of simulated cases. Conversely, customization triggered an adverse volumetric crowding effect in focal mononeuropathies, suggesting un-customized native auxetic foam as the preferred structural configuration for this specific cohort to avoid premature cell densification. Re-entrant cellular structures accommodate heterogeneous pathomechanics, offering an exploratory pathway for patient-specific orthotic optimization.

## 1. Introduction

Peripheral neuropathy remains a primary clinical driver of lower-extremity pathomechanics. This condition—characterized by nerve damage that disrupts sensory, motor, and autonomic functions—can originate from metabolic degradation in diabetic peripheral neuropathy (DPN), neurotoxic exposure in chemotherapy-induced peripheral neuropathy (CIPN), or focal structural damage from localized trauma [1,2]. The resulting loss of protective sensation results in unmitigated localized loading. Elevated Peak Plantar Pressures (PPP) during the stance phase of gait directly correlate with localized tissue ischemia, structural breakdown, and eventual ulceration. The assessment of these pathomechanical gait parameters relies heavily on the reliability and validity of high-resolution electronic pressure-measurement systems. These platforms isolate the spatial migration of peak localized stresses under dynamic conditions [3,4].

Traditional orthopedic insoles typically utilize viscoelastic materials, such as open-cell memory foams, or conventional closed-cell elastomeric foams. While conventional orthotic interventions focus on macro-scale load shifting using custom-molded elastomeric designs, managing specialized pathomechanics introduces unique material requirements. Under vertical compressive loading, conventional materials exhibit a positive Poisson’s ratio; they expand laterally away from the site of maximum force, which thins the material directly beneath vulnerable bony prominences. Conversely, thermo-mechanically processed foams exhibit an effective negative Poisson’s ratio. This macro-structural behavior arises directly from their re-entrant cellular architecture—wherein internal reticular ribs are deliberately buckled inward—rather than from the intrinsic molecular properties of the base polymer chemistry [5,6]. When subjected to a vertical compressive force, their internal re-entrant cell geometries contract inwardly, effectively concentrating mass directly beneath the point of loading to enhance local energy absorption and shock dissipation [5,6]. This specific structure-property-function relationship enables self-regulating local energy absorption, positioning re-entrant foams as an alternative to flat-profile conventional orthopedic substrates [7,8].

Managing specialized pathologies like CIPN introduces unique lower-extremity constraints. CIPN shifts baseline biomechanics, inducing migratory microvascular hyper-sensitivity and proprioceptive deficits without immediate skeletal structural collapse. Recent advancements in geometric optimization demonstrate that structural modifications to auxetic configurations can shift energy absorption thresholds, making them viable for advanced biomedical scaffolds and orthotic interventions [9,10]. Furthermore, the structural topology at the cell junctions—such as the integration of localized nodal spheres—plays a critical role in dictating the global mechanical response, stress distribution, and operational predictability of the auxetic substrate during physical deformation [11]. In particular, understanding how changes in geometric parameters influence core structural response provides a pathway for shifting energy absorption limits under localized force vectors [12], while optimizing nodal configurations directly improves the material predictability and operational reliability of cellular matrices under compressive loading [11].

In our baseline research, the preliminary structural behavior of flat, un-customized re-entrant foams was verified [13]. While our baseline research verified the uniform mechanical response of these foams, a major research gap remains in understanding how structural customization via modular inserts interacts with distinct neuropathic subsystems. Small pilot cohorts (*n* < 10) often violate standard parametric assumptions, obscuring true performance variance. The core contribution of this work is a unified data-driven orthotic pipeline that maps targeted geometric modifications against diverse pathomechanical signatures, evaluating whether material behaviors remain stable across varied patient profiles. This study addresses these limitations by deploying a standardized fabrication pipeline alongside a bootstrap resampling workflow (*B* = 1000) to evaluate the systemic stability of custom auxetic insoles across a heterogeneous cohort presenting distinct neuropathic subsystems. We specifically interrogate the mechanical trade-offs between native auxetic baselines and custom dome inserts, the hypothesized material break-in phenomenon, structural crowding constraints, and kinematic consistency during dynamic gait.

## 2. Materials and Methods

### 2.1. Material Synthesis and Insole Fabrication

The base auxetic orthotic substrates evaluated in this study were fabricated using a multi-stage patented thermo-mechanical transformation pipeline detailed extensively in previous configurations and intellectual property frameworks [14,15]. The underlying protocol leverages open-cell polyurethane (PU) precursor materials exhibiting a specialized composition and phase morphology optimized for structural stability. Operating under pre-calculated tri-axial compression parameters to enforce inward structural cellular folding, the multi-stage conversion sequence maps across: (1) a dedicated mold-loading phase to secure target volumetric compression ratios, (2) a specialized thermal processing phase executed above the softening temperature profile of the specific PU composition to drive stable structural reconfiguration, and (3) a room-temperature cooling phase to permanently fix the internal re-entrant struts [14,15]. To establish strict quantitative parity across the investigated insole blanks, all synthesized auxetic specimens used in the present exploratory trials were calibrated to match the uniform physical property profiles established in baseline investigations [13,16]: an operational thickness of 6 mm, a baseline density of 18.42 kg/m^3^, and an elastic modulus of 94 ± 5 kPa. This specific re-entrant configuration yields a stable negative Poisson’s ratio of −0.45, dynamically enabling the structural matrix to expand laterally under tension and contract under compression [10].

**Figure 1:**
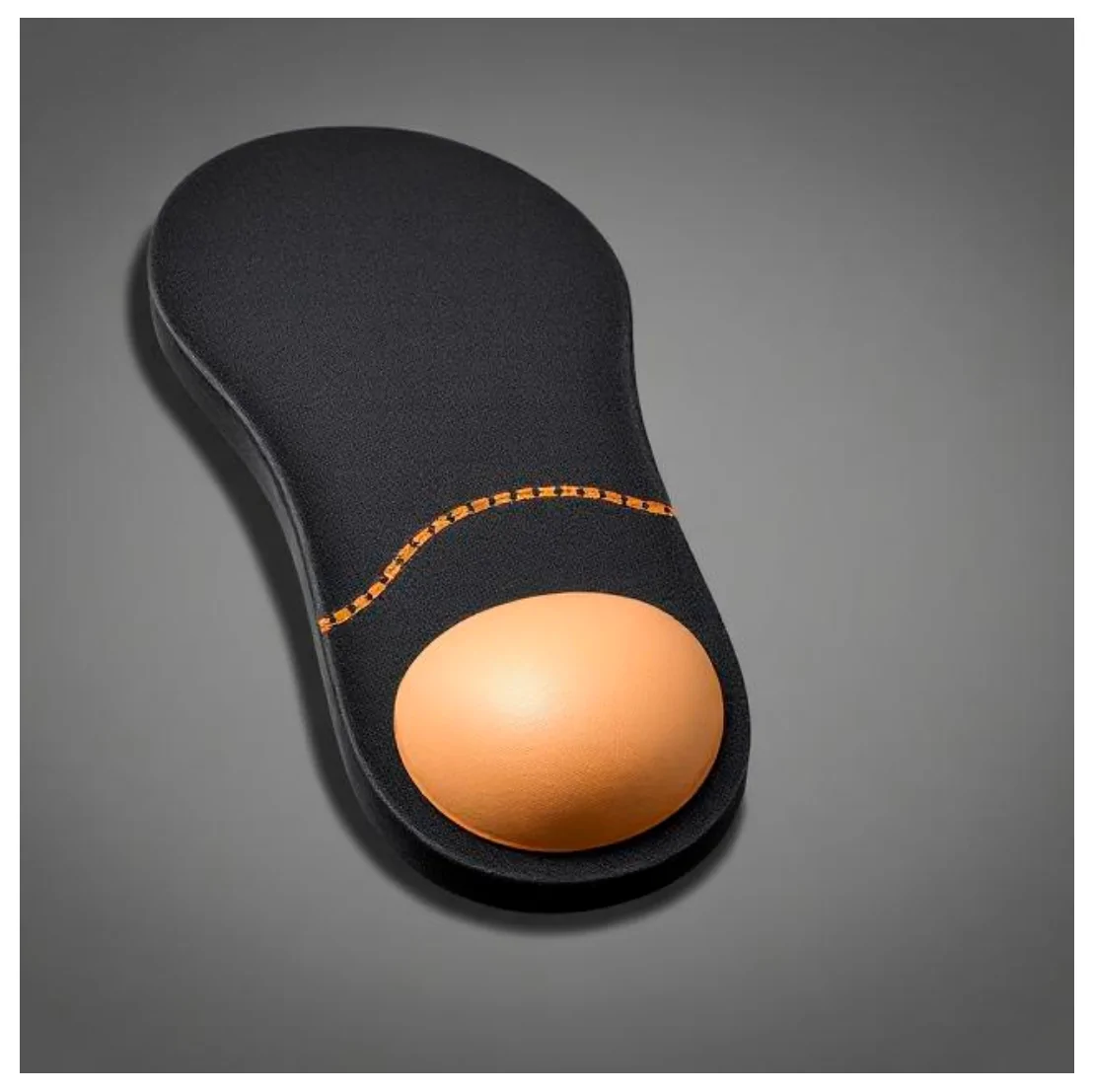
Schematic representation of the insole fabrication and representative assembly sequence. Localized placement of the custom auxetic dome varies across the cohort depending on the subject’s specific neuropathic etiology.

The custom fabrication pipeline was executed as follows:

1. Sizing and Cutting: Participant foot geometries were mapped to standard sizing bounds (US Sizes 6 through 11). Native auxetic foam blanks were precision-cut using standardized template configurations to ensure outer profile uniformity (Figure 1).
2. Adhesive Assembly: Rather than utilizing liquid bonding agents that risk infiltrating the open-cell network via capillary action and altering the localized hinge mechanics, the custom auxetic domes were secured to the native foam base layer using a high-tack, low-thickness double-sided adhesive tape film (Figure 1). This ultra-thin tape interface preserved structural continuity and allowed uninhibited lateral re-entrant cell contraction at the boundary layers during vertical compression.

**Figure 2:**
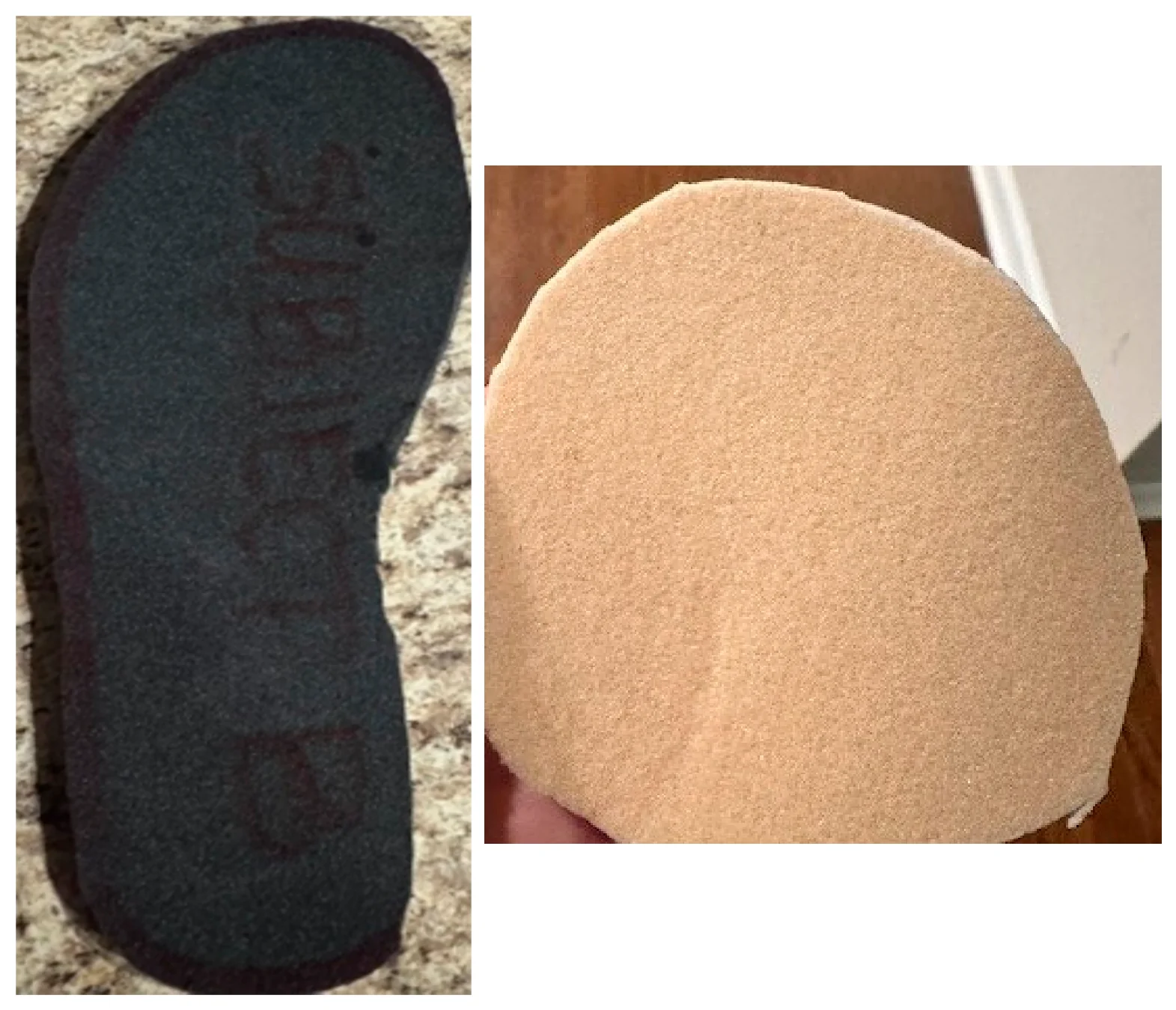
Modular components of the custom orthotic assembly, showing the un-customized native auxetic foam base insole (**left**; adapted from baseline configurations detailed in *Bioengineering* [13]) positioned alongside the newly integrated standalone modular auxetic dome add-on (**right**) prior to permanent assembly.

The localized pressure offloading relies on a physical pairing between this continuous orthotic foundation and the structural dome add-ons, which are customized to the individual subject’s specific empirical data prior to permanent application (Figure 2). To verify proper component integration before final multi-layer adhesion, the un-customized native auxetic foam base insole profile was evaluated directly alongside the independent standalone modular auxetic dome add-on configurations prior to permanent structural alignment assembly.

### 2.2. Etiology-Based Customization Framework

The heterogeneous nature of the clinical cohort required a structured framework to map specific pathological manifestations to targeted mechanical solutions. To address this, an empirical customization map was established (Table 1) to categorize the unique pathomechanical signatures and prescribe strategic insole sizing and configuration boundaries.

The structural configurations specified in Table 1 were developed and based on established podiatric, biomechanical, and tissue-ischemia literature [17,18,19,20,21]. Patients with DPN frequently present with intrinsic muscle wasting that shifts the natural adipose plantar fat pad distally, creating high vertical stress concentrations beneath isolated skeletal prominences [17,18]. Targeted high-density, low-profile auxetic domes were applied to these specific hubs to leverage the auxetic mass-concentration effect (v < 0) to dynamically buffer hyper-focal impact zones during peak stance. For CIPN, a moderate-profile midfoot auxetic wedge was integrated to fill the longitudinal arch, maximizing total plantar contact area and dissipating vertical shear stress across the entire midfoot matrix to safely account for post-chemotherapy nerve damage [19,20].

The high-magnitude loading from systemic obesity alters plantar pressure mechanics, accelerating anatomical fat-pad displacement and exposing deep bony structures to unmitigated vertical stress [21]. To compensate for this loss of shock absorption, a max-thickness, reinforced auxetic dome pairing was implemented across both major striking zones. Conversely, individuals presenting focal mononeuropathies from localized physical trauma (injury), exhibit rigid structural asymmetries or fixed bony lesions that require maximum material flexibility. Introducing extra volumetric material within the restricted confines of standardized footwear triggers a geometric crowding effect; this forces the re-entrant cellular ribs into premature self-contact, or densification, before the foot completes its roll-through, effectively locking out the material’s internal hinges. Retaining the un-customized, continuous native auxetic foam ensures that the underlying cellular structure remains free to buckle and flex naturally around rigid traumatic anomalies.

### 2.3. Participant Characteristics and Standardized Footwear Controls

To isolate the mechanical influence of the experimental orthotic inserts, and eliminate structural shoe variance during data collection, all shod testing conditions were executed using a standardized footwear model (Athletic Works basic running shoes; Walmart Apollo, LLC, Bentonville, AR, USA). This conventional flat-profile memory foam bed serves as an authentic, high-utility baseline representing the entry-level viscoelastic cushioning configurations frequently utilized by neuropathic individuals for real-world self-management, thereby isolating how an effective negative Poisson’s ratio structure mechanically shifts peak localized load under identical footwear boundaries.

The stock conventional memory foam insoles pre-installed in the commercial shoes were completely removed prior to testing and initially utilized as physical geometric sizing templates to guide the manual cutting and profile matching of the auxetic insoles. During the dynamic walking protocols, participants were evaluated under four comparative conditions: barefoot, utilizing the factory-installed flat memory foam insoles re-inserted into the shoe enclosures to serve as the designated over-the-counter (OTC) control condition, and utilizing the experimental native and/or custom auxetic insoles placed inside the same standardized shoe model. This strict standardization tracking ensured that any observed variations in peak plantar pressure distribution were uniquely a function of the dynamic interface between the user’s foot, the specific orthotic geometry, and the baseline pathology, rather than confounding structural artifacts from varied personal footwear designs.

A pilot cohort of nine participants (*N* = 9, 5 females, 4 males) was recruited through the National Aeronautics Space Administration (NASA)/Marshall Space Flight Center (MSFC). The cohort comprised seven subjects presenting with confirmed peripheral neuropathy or high loading states: metabolic diabetes (*n* = 2), localized traumatic physical injury (*n* = 2), oncological treatment-induced neurotoxicity *(n* = 2), and high-magnitude global mechanical loading (*n* = 1). Two healthy individuals with no neuropathic attributes were retained as controls. These healthy controls were retained in all quantitative cohort analyses to preserve methodological symmetry across testing conditions and to provide a descriptive benchmark for standard walking mechanics, rather than for direct clinical comparison. The complete cohort presented a mean age of 47.33 ± 9.63 years and a mean body mass of 211.11 ± 66.8 lbs. All participants provided written informed consent under testing protocols reviewed and approved by the NASA Institutional Review Board (IRB) (Johnson Space Center, Houston, TX, USA). Complete baseline demographics, footwear histories, and subjective post-trial outcomes with the auxetic foam inserts are detailed in Table 2.

### 2.4. Gait Analysis

**Figure 3:**
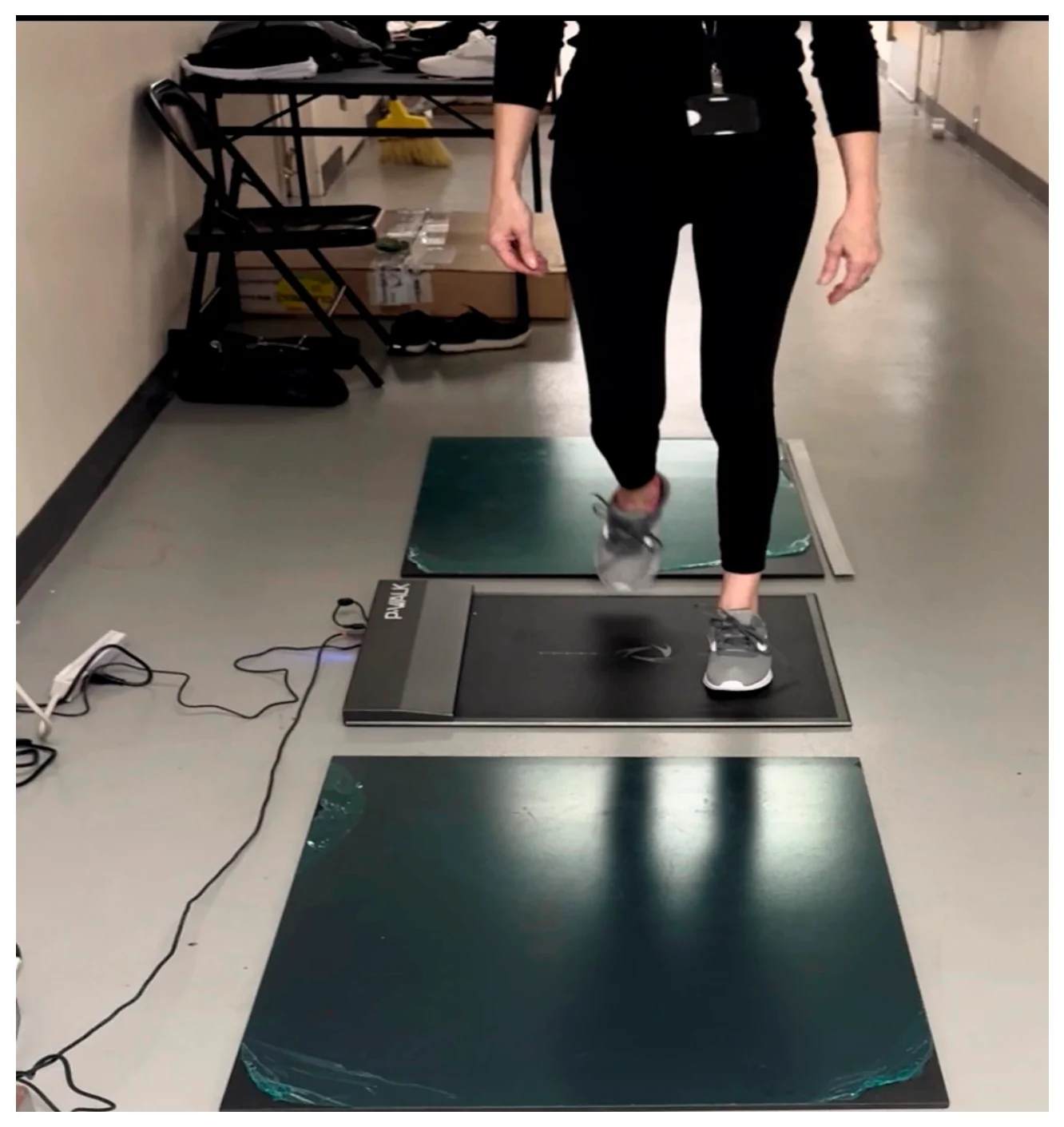
A photograph of the experimental configuration illustrating the synchronized electronic pressure platform.

Bilateral gait assessments were conducted utilizing the P-Walk 600 high-resolution electronic pressure platform (BTS Bioengineering S.p.A., Milan, Italy) (Figure 3) [22]. Participants executed multiple walking trials at a self-selected, comfortable cadence over the sensor matrix under four primary conditions: barefoot, OTC, un-customized native auxetic foam, and custom-dome auxetic insoles. Kinematic trends were monitored simultaneously using a MARVUE 2D motion capture system (Shenzhen Guantu Technology Co., Ltd., Shenzhen, China). The healthy controls did not walk with the customized auxetic insoles, as custom dome placement was strictly dictated by the presence of pathological high-pressure etiology zones.

The primary quantitative outcome metric extracted across all testing configurations was the average bilateral PPP, converted from pounds per square inch (psi) to kilopascals (kPa) (Table A1), across the anatomical zones. This is mathematically defined as

$$PPP_{avg}=\frac{\begin{array}{c} PPP_{Left}+\\ PPP_{Right} \end{array}}{2}$$

This formula represents the mean of the maximum localized pressure values captured across valid gait trials.

## 3. Results

### 3.1. Material Conformity

Initial gait trials revealed a distinct mechanical behavior unique to the auxetic structures. For the majority of the neuropathic cohort, the un-customized native auxetic foam initially yielded higher PPPs compared to the OTC controls. This behavior is attributed to a hypothesized structural cell-adaptation (break-in) phase where localized re-entrant cells undergo initial compressive cyclic loading before reaching stable volumetric conformity to the wearer’s unique plantar morphology.

### 3.2. Etiology-Specific Customization vs. Native Performance

**Figure 4:**
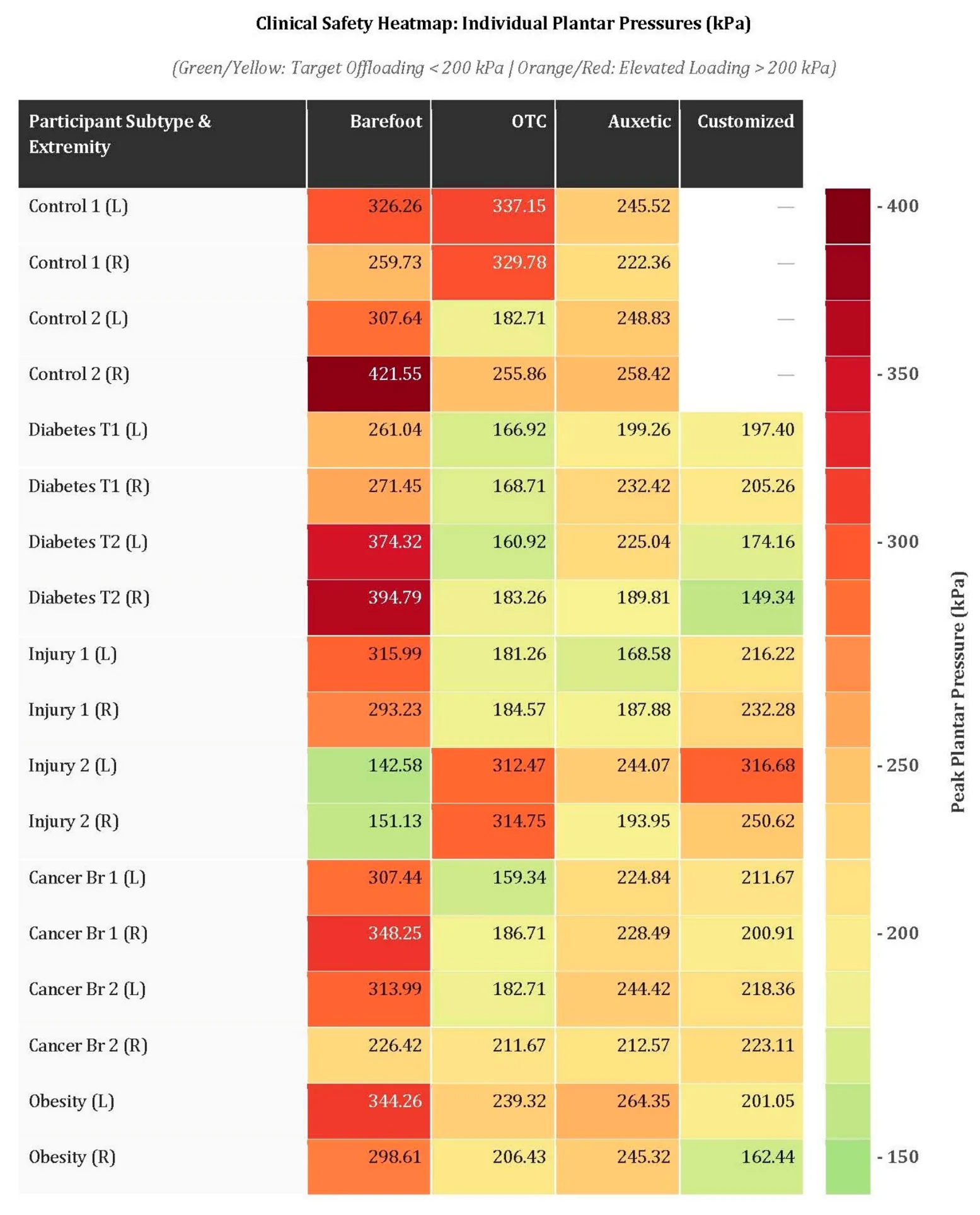
Spatial load distribution matrix across the individual cohort under the four testing configurations. Green/yellow indicates values below or near the exploratory safety target threshold (<200 kPa), while dark orange/red denotes elevated loading zones (>200 kPa).

Spatial load distribution across the individual plantar regions was mapped using a dynamic Clinical Safety Heatmap matrix (Figure 4). Figure 4 contains both healthy controls and symptomatic subjects to illustrate relative shifts across all recorded testing boundaries.

**Figure 5:**
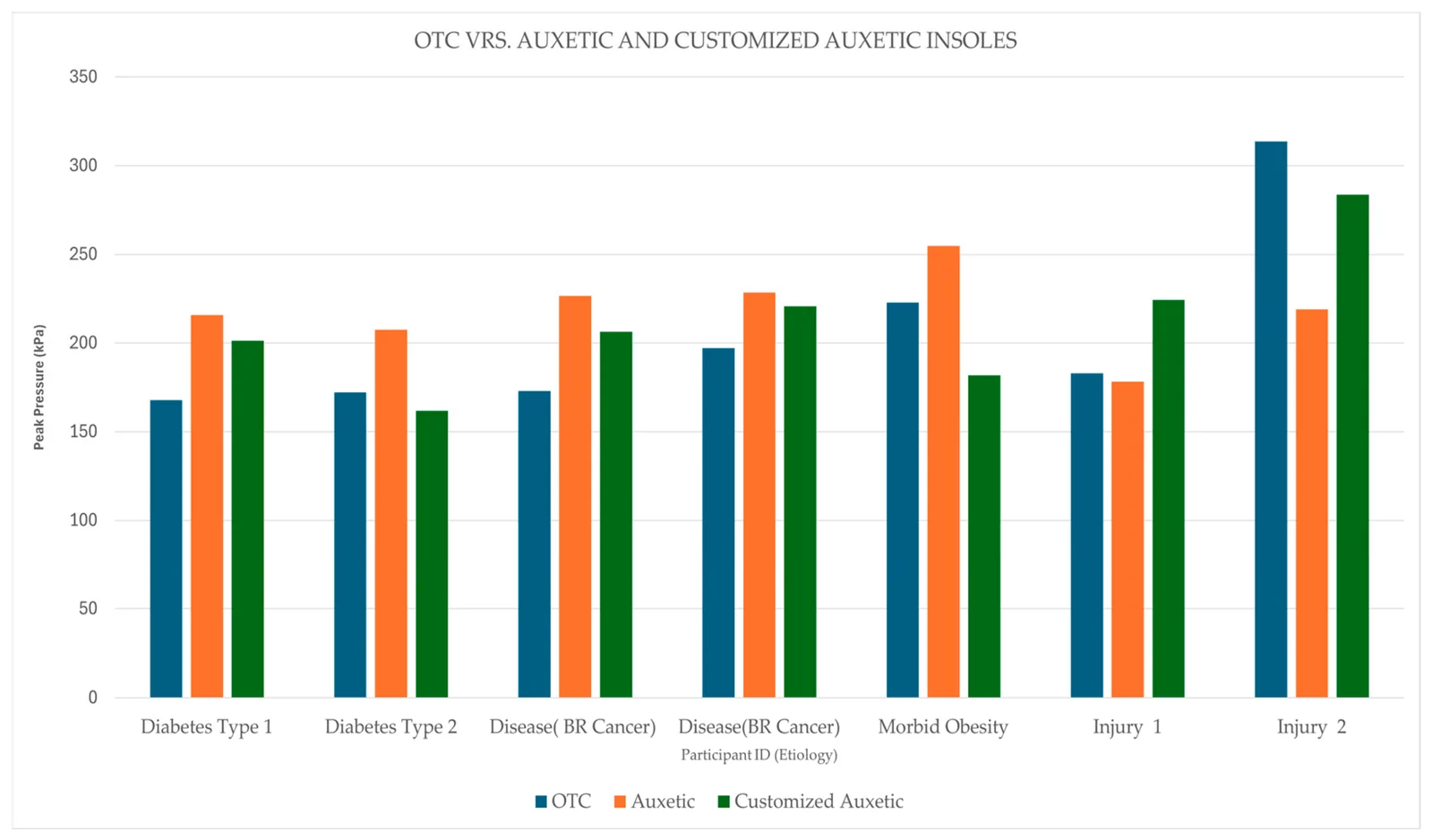
Comparative analysis of average bilateral peak plantar pressures across symptomatic neuropathic subgroups under varied orthotic conditions.

To further quantify the comparative performance between standard commercial interventions and the auxetic models, PPP(kPa) was plotted across the symptomatic sub-group (see Figure 5). Healthy controls are omitted from Figure 5 because they did not receive highly individualized custom dome interventions, as custom configurations were strictly mapped to pathological high-pressure etiologies.

The performance trajectories captured across the comparative metrics demonstrate that structural outcomes were contingent upon the pre-planned interventions mapped in Table 1:

- Systemic Polyneuropathies (DPN/CIPN/Obesity)**:** Customized auxetic insoles optimized load profiles across systemic subjects (Figure 4). In the Type 2 Diabetes and Obesity subgroups, the customized auxetic framework succeeded in lowering localized loading beneath both the OTC and native auxetic baselines, achieving a peak pressure reduction of up to 62.2% (dropping from a barefoot baseline of 394.79 kPa down to 149.34 kPa under the custom auxetic intervention) compared to barefoot trials (Figure 4). This shifted the loading signature beneath the clinical safety target threshold of <200 kPa in 55% of the recorded systemic cases (Figure 4).
- Injury-Induced Mononeuropathies: In participants with localized injury-induced neuropathy, an inverse performance profile was prevalent (Figure 4 and Figure 5). While the native auxetic foam decreased PPP relative to OTC controls in the Injury 2 cohort (dropping pressure from >300 kPa down to approximately 220 kPa) (Figure 4 and Figure 5), the addition of the customized dome inserts led to a crowding effect. This localized volumetric excess spiked pressures back up near 285 kPa (Figure 4 and Figure 5), suggesting that the un-customized native auxetic foam represented the preferred configuration for localized mononeuropathies within this small cohort by preventing premature cellular densification.

**Figure 6:**
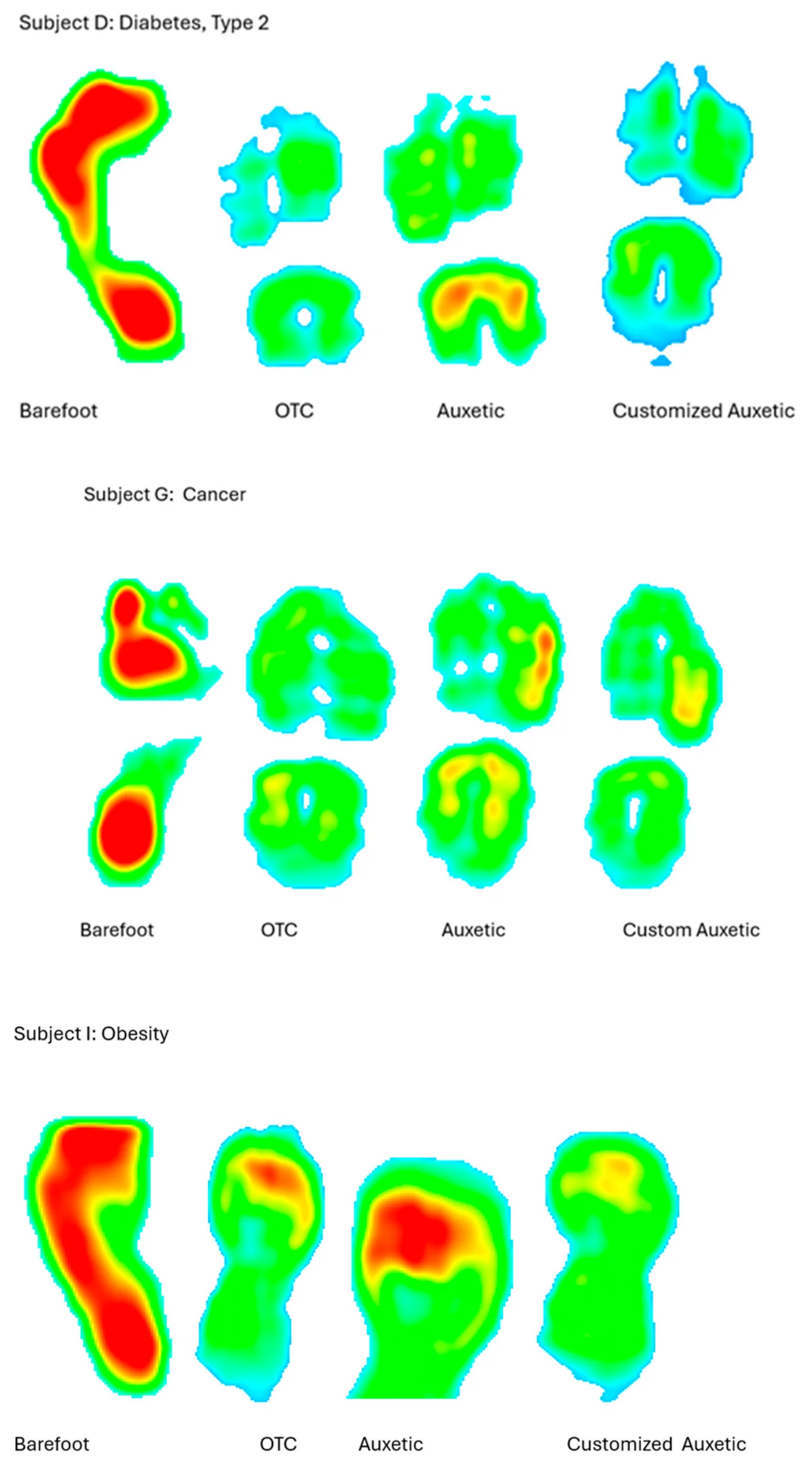
Representative baropodometric plantar pressure distribution signatures across systemic neuropathies and metabolic conditions: (D) DPN, (G) CIPN, and (I) Metabolic Obesity. All panels utilize a standardized, normalized color-pressure scale bounded from 0 kPa (dark blue/clear) to 450 kPa (crimson red) to guarantee visual and quantitative parity across experimental configurations.

**Figure 7:**
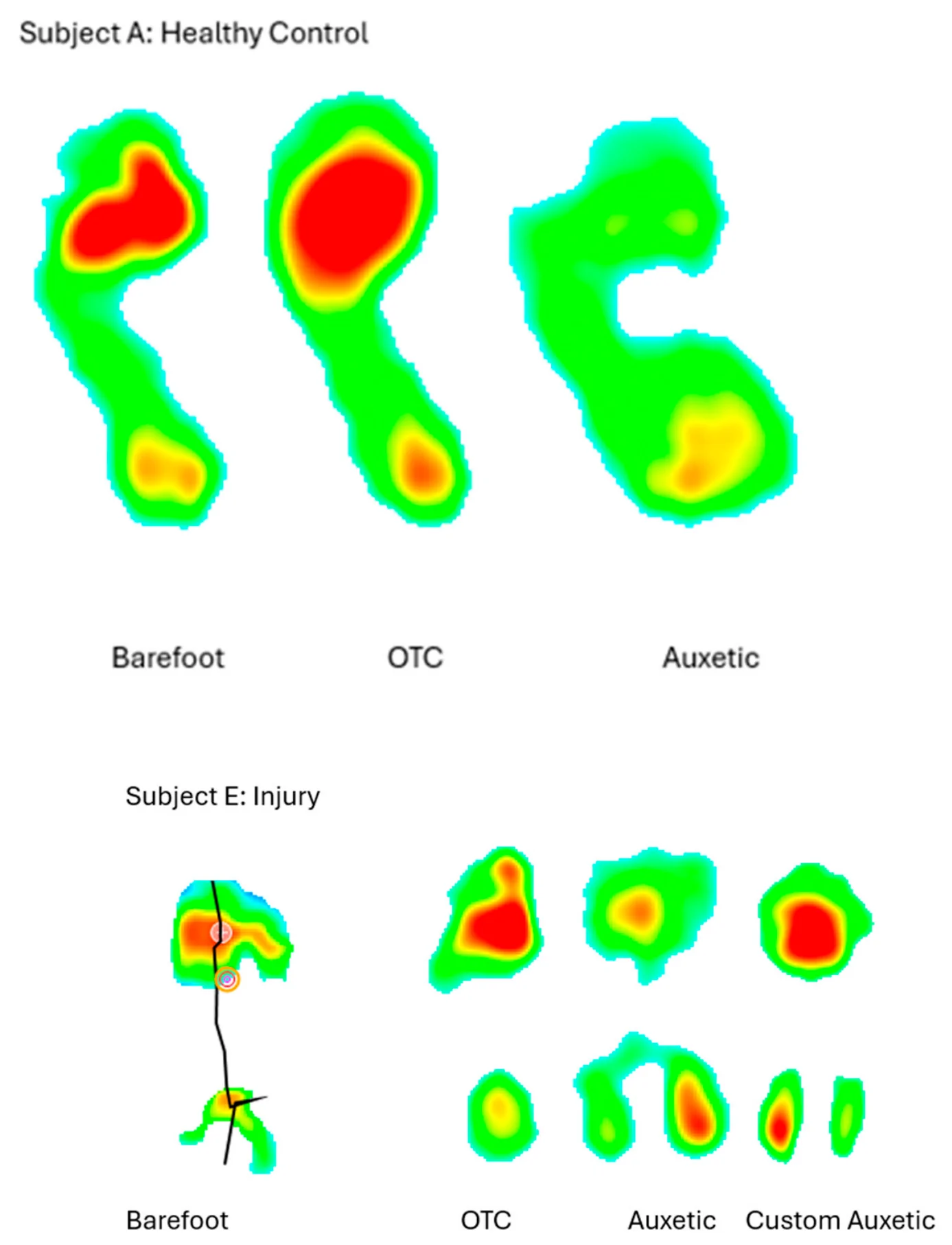
Comparative baropodometric pressure mapping of a baseline healthy control across three standard conditions (**top**) and Subject E (injury-induced neuropathy) across four testing configurations (**bottom**), highlighting the development of the localized geometric crowding phenomenon. All panels utilize a standardized, normalized color-pressure scale bounded from 0 kPa (dark blue/clear) to 450 kPa (crimson red) to guarantee visual and quantitative parity across experimental configurations. The solid black trajectory line denotes the Center of Pressure (CoP) path during gait, while the intersected concentric circular symbols highlight localized peak focal hotspots.

Baseline barefoot pressure profiles across all symptomatic subjects revealed severe, localized plantar pressure concentrations, concentrated primarily at the first metatarsal head and the calcaneus. To visually demonstrate these localized tracking dynamics, representative baropodometric pressure mappings are presented in Figure 6 and Figure 7. Both figures utilize a standardized, normalized color-pressure scale bounded from 0 kPa (dark blue/clear) to 450 kPa (crimson red) to guarantee visual and quantitative parity across configurations.

Plantar pressure distribution signatures across the systemic neuropathies and metabolic conditions—including diabetic peripheral neuropathy (Subject D), chemotherapy-induced peripheral neuropathy (Subject G), and metabolic obesity (Subject I)—are illustrated in Figure 6. Across these systemic groups, the customized geometric configurations achieved predictable offloading profiles, expanding total plantar contact area and driving localized stress values beneath baseline thresholds.

The healthy control panels in Figure 7 illustrate a normative load propagation profile across barefoot, OTC, and native auxetic conditions. As established in electronic baropodometric literature, this normative benchmark signature is characterized by a uniform, progressive load roll-through that graduates smoothly from the calcaneus, propagates cleanly along the structural lateral midfoot border, and distributes across the anterior metatarsal heads [4]. In contrast to the positive systemic loading trends in Figure 6, the loading profile for Subject E (injury-induced neuropathy) (Figure 7) demonstrates the unique pathomechanical impact of local structural trauma. While the native auxetic baseline reduces peak pressure focus relative to the OTC condition for Subject E, the application of the customized geometric designs introduces unexpected biomechanical variations. Under this restricted structural constraint, the custom dome insert triggers an adverse geometric crowding phenomenon that concentrates vertical stress back into a prominent focal peak.

Despite these localized pressure variations, motion capture data acquired via the MARVUE 2D system (Shenzhen Guantu Technology Co., Ltd., Shenzhen, China) confirmed observed qualitative trends toward stabilized gait paths and consistent roll-through cycles across the pilot cohort when utilizing auxetic materials compared to barefoot trials.

### 3.3. Computational Verification via Robust Bootstrap Resampling

The statistical behavior of the structural load distribution was confirmed through 1000 bootstrap resampling iterations which were performed using XLSTAT (version 2026.1, Lumivero, Denver, CO, USA) (Table 3). The native auxetic foam exhibited mechanical reliability across diverse subject profiles, maintaining a Consistency Rank of 1—demonstrating that within the simulated data configurations, the native auxetic foam regularly maintained a more tightly bound load distribution compared to the OTC condition. Furthermore, the bootstrapped baseline PPP across the 72 fully populated data vectors converged at an overall mean of 32.873 kPa, and exhibited a stable bootstrap standard deviation (σ*) of 12.063 kPa [23] (Table 3).

Variables such as “Insole Variant Grouping” and “Neuropathy Subsystem Type” are included in Table 3 strictly to document the structural composition and nominal vector scaling of the resampled matrix data blocks, ensuring methodological transparency. The narrow Confidence Interval (CI) captured for the PPP distribution demonstrates that the mechanical response of the re-entrant auxetic structures remains stable within this localized observation pool.

## 4. Discussion

The experimental data and subsequent bootstrap simulation confirm that the custom thermo-mechanical fabrication protocol produces a consistent orthotic, extending the feasibility bounds established in our previous work [13]. While our prior pilot was strictly limited to evaluating uniform, un-customized flat auxetic sheets to verify basic material feasibility, the present work introduces a novel etiology-driven custom fabrication framework to uncover mechanical boundaries—such as the cellular densification thresholds driving the geometric crowding phenomenon—that cannot be observed using flat sheets alone.

Our findings demonstrate that the inward lateral contraction (negative Poisson’s ratio effect) of the re-entrant cell matrix provides a self-regulating buffering zone. When a concentrated vertical load is applied, the local cells draw together, resulting in an increase in local density and thickness directly under that specific force vector. The data also highlights a vital design paradigm: customization is not universally additive. For systemic polyneuropathies, where widespread sensory degradation requires broad load shifting, the spatial redistribution provided by custom dome inserts justifies the increased material volume.

For localized trauma, however, introducing excess material volume into a spatially constrained footwear environment limits the available strain boundary layers. This triggers a geometric crowding phenomenon: under vertical load, the re-entrant cell structures reach their maximum compressive strain early in the gait cycle. This forces the internal cell struts into premature self-contact—the densification region—effectively transforming the compliant foam into a highly rigid solid block that spikes local pressure, rendering un-customized native foam an important exploratory preference for localized anomalies.

Additionally, the target clinical threshold of <200 kPa used in this study must be interpreted with caution. While podiatric consensus leverages this value as a reliable indicator for mitigating tissue ischemia [24], absolute values remain highly system-dependent across different sensor array interfaces [25]. Thus, maintaining pressures beneath 200 kPa represents an exploratory safety target rather than an absolute clinical guarantee against skin breakdown.

### Limitations and Future Research

A significant limitation of this pilot exploration is the lack of strict gait velocity constraints. Because walking speed directly modulates plantar pressure distributions [26,27], unmetered velocity variations may introduce confounding artifacts that influence pressure sensitivity. Furthermore, the cohort size (*N* = 9) was intended for pilot observation; therefore, the results should be validated through larger, multi-center clinical trials to ensure broad generalizability across more diverse patient populations. Future research must specify the temporal duration and exact histological type of these clinical etiologies to refine the orthotic customization. Finally, future studies should evaluate the mechanical stability and fatigue life of the auxetic foam structures under long-term daily usage conditions.

## 5. Conclusions

This research demonstrates that etiology-specific mapping of auxetic foams offers a useful exploratory framework for orthotic design, validating the preliminary technical feasibility of custom-fabricated auxetic foam insoles for plantar pressure redistribution. Subjects presented with systemic polyneuropathies benefited from tailored customization, reaching the exploratory <200 kPa clinical safety target threshold in 55% of the evaluated simulated cases. Conversely, for localized structural injuries, preliminary observations suggest that the native auxetic configuration remains a preferred choice due to its uninhibited re-entrant structure, which provides sufficient localized shock absorption without exacerbating pressure sensitivity through added material volume.

Rather than establishing definitive clinical guidelines or absolute contraindications, this work highlights exploratory mechanical trends that justify the pursuit of larger-scale, rigorously controlled clinical trials. Future studies employing standardized walking velocity protocols and objective kinematic assessments are necessary to definitively characterize the mechanical influence of auxetic technology in rehabilitative applications.

## Figures and Tables

**Table 1:** Empirical Customization Map: PPP Patterns and Strategic Auxetic Interventions.

Neuropathic/Loading Subsystem	Primary Pathomechanical PPP Signature	Sizing Boundaries	Strategic Customization Intervention Matrix
Diabetic Peripheral Neuropathy (DPN)	Severe focal stress at the 1st metatarsal head and calcaneus; high ulcer risk.	Sizes 8.5–10	Target high-density, low-profile custom auxetic dome inserts at primary pressure hubs.
Chemotherapy-Induced Neuropathy (CIPN)	Diffuse, migratory microvascular hyper-sensitivity across the midfoot and digits.	Sizes 10–11	Moderate-profile midfoot auxetic wedge integration with soft top-layer pairing.
Localized Physical Trauma (Injury)	Rigid structural asymmetries; high fixed unilateral plantar loading over bony lesions.	Sizes 6.5–7	Exploratory Trend: Retention of un-customized, continuous native auxetic foam suggested.
Systemic Global Mechanical Loading (Obesity)	High-magnitude global vertical loading with rapid heel/forefoot fat pad displacement.	Size 11W	Max-thickness, reinforced auxetic dome pairing across both heel and forefoot zones.

Note: Empirical customization map pairing primary neuropathic pathomechanical signatures with strategic auxetic orthotic interventions.

**Table 2:** Cohort Demographics, Baseline Footwear, and Subjective Ambulation Outcomes.

Participant Etiology Subtype	Anthropometrics (Age, Sex, Wt., Shoe Size)	Baseline Footwear Worn to Site	Subjective Comfort vs. Baseline	Perceived Gait vs. Baseline
Subject A Control 1 (Healthy)	45/F/180 lbs/8	Old Navy Canvas Shoes	More comfortable	About the same
Subject B Control 2 (Healthy)	44/F/240 lbs/8W	New Balance 1540 B	More comfortable	Walking is better
Subject C Diabetes T1 (Systemic)	59/F/118 lbs/8.5	New Balance 2010 B	More comfortable	Walking is better
Subject D Diabetes T2 (Systemic)	45/M/210 lbs/10	Adidas Lightshift B	More comfortable	Walking is better
Subject E Injury 1 (Focal)	59/F/173 lbs/6.5W	Under Armour Running Shoes	More comfortable	Walking is better
Subject F Injury 2 (Focal)	61/F/146 lbs/7	Merrell Alpine 83	About the same	Walking is better
Subject G Cancer Br 1 (Systemic)	40/M/270 lbs/10W	Skechers Slip-ins: Glide-Step	More comfortable	Walking is better
Subject H Cancer Br 2 (Systemic)	38/M/173 lbs/11	Brooks Adrenaline/Curex Insole	About the same	About the same
Subject I Obesity (Metabolic)	35/F/330 lbs/11W	New Balance Fresh Foam X	More comfortable	Walking is better

Note: Summary of baseline participant anthropometrics (age, sex, weight, shoe size), historical footwear configurations, and comparative subjective ambulation outcomes following the auxetic foam interventions.

**Table 3:** Bootstrapped Target Plantar Pressure & Systemic Distribution Metrics (*B* = 1000).

Evaluated Parameter Matrix	Valid Simulated N	Absolute Minimum	Absolute Maximum	Bootstrapped Cohort Mean	Bootstrap Standard Deviation (σ*)	95% Bootstrap Confidence Interval
Insole Variant Grouping	72	1	4	2.472	1.113	—
Neuropathy Subsystem Type	72	1	9	5	2.6	—
Peak Plantar Pressure (PPP)	72	0	61.14	32.873 kPa	12.063 kPa	[30.09 kPa, 35.66 kPa]

Note: Analysis performed on trial-averaged profiles (*n* = 9 subjects; simulated matrices *N* = 72). Values denote scale boundaries, converged means, and the true bootstrap standard deviation (σ*). Effect sizes (r) utilize a denominator of *n* = 9 to strictly reflect true inter-subject variance and prevent pseudoreplication (magnitudes: small = 0.1, medium = 0.3, large = 0.5).

## Data Availability

The original contributions presented in this study are included in the article. Further inquiries can be directed to the corresponding author.

## Abbreviations

The following abbreviations are used in this manuscript:

BMI	Body Mass Index
CIPN	Chemotherapy-Induced Peripheral Neuropathy
DPN	Diabetic Peripheral Neuropathy
IRB	Institutional Review Board
kPa	Kilopascals
NASA	National Aeronautics Space Administration
PPP	Peak Plantar Pressure
PSI	Pounds per Square Inch

## Appendix A

**Table A1:** Participant-specific peak plantar pressure values (PSI/kPa) across testing conditions.

Group	Foot	Barefoot (psi/kPa)	OTC (psi/kPa)	Auxetic (psi/kPa)	Customized Auxetic (psi/kPa)
Control	L	47.32/326.26	48.90/337.15	35.61/245.52	—
	R	37.67/259.73	47.83/329.78	32.25/222.36	—
Control	L	44.62/307.64	26.50/182.71	36.09/248.83	—
	R	61.14/421.55	37.11/255.86	37.48/258.42	—
Diabetes (T1)	L	37.86/261.04	24.21/166.92	28.90/199.26	28.63/197.40
	R	39.37/271.45	24.47/168.71	33.71/232.42	29.77/205.26
Diabetes (T2)	L	54.29/374.32	23.34/160.92	32.64/225.04	25.26/174.16
	R	57.26/394.79	26.58/183.26	27.53/189.81	21.66/149.34
Injury 1	L	45.83/315.99	26.29/181.26	24.45/168.58	31.36/216.22
	R	42.53/293.23	26.77/184.57	27.25/187.88	33.69/232.28
Injury 2	L	20.68/142.58	45.32/312.47	35.40/244.07	45.93/316.68
	R	21.92/151.13	45.65/314.75	28.13/193.95	36.35/250.62
Cancer (Br)	L	44.59/307.44	23.11/159.34	32.61/224.84	30.70/211.67
	R	50.51/348.25	27.08/186.71	33.14/228.49	29.14/200.91
Cancer (Br)	L	45.54/313.99	26.50/182.71	35.45/244.42	31.67/218.36
	R	32.84/226.42	30.70/211.67	30.83/212.57	32.36/223.11
Obesity	L	49.93/344.26	34.71/239.32	38.34/264.35	29.16/201.05
	R	43.31/298.61	29.94/206.43	35.58/245.32	23.56/162.44

Note: Participant-specific peak plantar pressure distributions (psi/kPa) across testing conditions. This dataset provides the raw measurements utilized for the bootstrap resampling analysis described in Section 3.3.
